# Saliva as alternative to naso-oropharyngeal swab for SARS-CoV-2 detection by RT-qPCR: a multicenter cross-sectional diagnostic validation study

**DOI:** 10.1038/s41598-022-16849-1

**Published:** 2022-07-23

**Authors:** Michael L. Tee, Aedrian A. Abrilla, Cherica A. Tee, Leslie Michelle M. Dalmacio, Vivencio Jose P. Villaflor, Al-Zamzam A. Abubakar, Pedrito Y. Tagayuna, Sheldon Steven C. Aquino, Vicente Aaron L. Bernardo, Ronald R. Matias

**Affiliations:** 1 College of Medicine and Philippine General Hospital, University of the Philippines Manila, Pedro Gil Street, Ermita, 1000 Manila, National Capital Region Philippines; 2 Medical Center Manila, Manila, National Capital Region Philippines; 3 Dagupan Doctors Villaflor Memorial Hospital, Dagupan City, Pangasinan Philippines; 4 Ciudad Medical Zamboanga, Zamboanga City, Zamboanga del Sur Philippines; 5 Fe Del Mundo Medical Center, Quezon City, National Capital Region Philippines; 6 St. Luke’s Medical Center, National Capital Region, Quezon City, Philippines

**Keywords:** Biotechnology, Microbiology, Molecular biology, Health care, Medical research, Molecular medicine

## Abstract

Saliva has been demonstrated as feasible alternative to naso-oropharyngeal swab (NOS) for SARS-CoV-2 detection through reverse transcription quantitative/real-time polymerase chain reaction (RT-qPCR). This study compared the diagnostic agreement of conventional NOS, saliva with RNA extraction (SE) and saliva without RNA extraction (SalivaDirect) processing for RT-qPCR in identifying SARS-CoV-2. All techniques were also compared, as separate index tests, to a composite reference standard (CRS) where positive and negative results were defined as SARS-CoV-2 detection in either one or no sample, respectively. Of 517 paired samples, SARS-CoV-2 was detected in 150 (29.01%) NOS and 151 (29.21%) saliva specimens. The saliva-based tests were noted to have a sensitivity, specificity and accuracy (95% confidence interval) of 92.67% (87.26%, 96.28%), 97.55% (95.40%, 98.87%) and 96.13% (94.09%, 97.62%), respectively, for SE RT-qPCR and 91.33% (85.64%, 95.30%), 98.91% (97.23%, 99.70%) and 96.71% (94.79%, 98.07%), respectively, for SalivaDirect RT-qPCR compared to NOS RT-qPCR. Compared to CRS, all platforms demonstrated statistically similar diagnostic performance. These findings suggest that both conventional and streamlined saliva RT-qPCR are at least non-inferior to conventional NOS RT-qPCR in detecting SARS-CoV-2.

## Introduction

Integral to the control of the coronavirus disease 2019 (COVID-19) pandemic is accurate, adequate and timely detection of severe acute respiratory syndrome coronavirus 2 (SARS-CoV-2) infections^1^. Identification of specific combinations of SARS-CoV-2 ribonucleic acid (RNA) genomic elements through reverse transcription quantitative/real-time polymerase chain reaction (RT-qPCR) has been the internationally accepted reference testing platform to this end, with naso-oropharyngeal swab (NOS) being the most commonly preferred patient sample^2^. As the algorithm from sample collection to testing in this prevailing paradigm is rife with economic, logistical and personnel safety issues^3,4^, we need to establish evidence-based, streamlined workflow that maintains reasonable diagnostic accuracy. This gains more importance in resource-disadvantaged developing nations that may disproportionately bear the brunt of the pandemic, such as the Philippines. With a peak test positivity rate of almost 50%, the country has more than 3.6 million total cases and over 60,000 deaths^5^. It is second only to Indonesia in terms of absolute COVID-19 burden among Southeast Asian nations^6^.

The evidence of saliva having diagnostic utility that is equivalent to NOS for detecting SARS-CoV-2 has since grown substantially to professional and public acceptance^7,8^. Considering the potential to additionally reduce local testing costs and requirements, we endeavored to comprehensively evaluate conventional saliva processing and SalivaDirect^9^, an RNA extraction-free saliva sample processing technique, against conventional NOS as sample preparation approach for detecting SARS-CoV-2 through RT-qPCR in the Philippine setting.

## Materials and methods

### Study design and ethics review

The protocol of this prospective cross-sectional study was reviewed and approved, with its implementation monitored, by the ManilaMed Ethics Review Committee (MMERC No. 2021-06). The study was performed in accordance with the Declaration of Helsinki and the International Ethical Guidelines for Biomedical Research involving human subjects.

### Participants

Volunteers considered potentially eligible for inclusion and eventually invited to participate, through consecutive sampling, were inpatient or outpatient adults availing NOS RT-qPCR SARS-CoV-2 testing, from May to December 2021, in one of three Philippine hospitals: Fe Del Mundo Medical Center (Quezon City, National Capital Region), Dagupan Doctors Villaflor Memorial Hospital (Dagupan City, Pangasinan Province, Luzon) and Ciudad Medical Zamboanga (Zamboanga City, Zamboanga del Sur, Mindanao). The volunteers were included if they are able to give informed consent and to autonomously collect drooled saliva. Volunteers were excluded if they were unable to ensure avoidance of enteral intake, gargling with mouthwash or brushing teeth, and smoking for at least 30 min before providing saliva. Demographic and clinical data such as history of exposure and symptoms were obtained from consenting participants.

### Test methods

Paired samples of saliva and NOS were collected from all volunteers. The samples of each patient were placed in separate code-labeled tubes (one for NOS and one for saliva), and these tubes were stored for transport in containers distinctively carrying only either type of sample. These were subjected to subsequent laboratory procedures within 24 h from sample collection, with separation in terms of personnel and instruments based on sample type. Interpretation of the tests was done independently by two assessors who did not have access to clinical information, and the result from the counterpart sample of the same study volunteer. The samples were processed and tested in laboratories using harmonized protocols in the aforementioned hospitals that are licensed to operate SARS-CoV-2 RT-qPCR facilities by the Philippine Department of Health.

#### Saliva RT-qPCR

Prior to NOS sampling, included volunteers were asked to drool at least 1 mL of saliva in a 5 mL sterile tube. The filled sterile tube was then sealed and stored in a chest at room temperature before transport to the designated laboratory. Each saliva sample was partitioned for conventional saliva RNA extraction (SE) and SalivaDirect procedures. The volume allotted for SE was subjected to manufacturer-prescribed conditions through the Nextractor NX-48 automated system (Genolution Inc., South Korea). Sample preparation for SalivaDirect was performed following the method described by Vogels et al.^9^. In brief, 2.5 μL (50 mg/mL) of Proteinase K was added to 50 μL saliva in PCR tubes, which were then vortexed at 3200 revolutions per minute for 1 min. The samples were then heated at 95 °C for 5 min. From these RNA-extracted and SalivaDirect-processed mixtures, 5 μL was utilized as input in subsequent RT-qPCR processes to amplify SARS-CoV-2 RNA-dependent RNA polymerase (*RdRp*), envelope (*E*), and nucleocapsid (*N*) genes using the GeneFinder COVID-19 Plus RealAmp Kit (OSANG Healthcare Co., Ltd., South Korea). The kit utilizes human *RNase P* gene template as internal control, and *RdRp*, *E*, *N* and human *RNase P* amplified constructs in RT-qPCR are dyed with fluorescein amidite (FAM), Texas Red, 5'-dichloro-dimethoxy-fluorescein/Victoria (JOE/VIC), and Cy5 fluorophores, respectively. An RT-qPCR run was deemed valid if (1) the quantification cycle (C_q_) readings for *RdRp*, *E*, *N* and *RNase P* were all ≤ 22.00 for the designated positive controls and (2) the negative control C_q_ readings for the same genes were all ≥ 40.00 or blank/undetermined. A sample was considered positive for SARS-CoV-2 if the C_q_ readings for *RNAse P* and at least either *RdRp*, *E* or *N* were ≤ 40.00, with the characteristic sigmoidal amplification curve noted in all instances. A negative result for SARS-CoV-2 was indicated if *RNase P* C_q_ ≤ 40.00 with sigmoidal amplification curve and C_q_ readings for *RdRp*, *E* and *N* were all ≥ 40.00 or blank/undetermined. Sample retesting was immediately performed if *RNase P* C_q_ ≥ 40.00 regardless of *RdRp*, *E* and *N* C_q_ values. All test reactions were maintained in the CFX96 RT-qPCR system (Bio-Rad Laboratories, California, United States of America).

#### NOS RT-qPCR

The conventional RNA extraction-dependent RT-qPCR technique to detect SARS-CoV-2 in NOSs was utilized to evaluate the utility of the saliva-based tests. Volunteers underwent naso-oropharyngeal swabbing performed by trained personnel, and the swabs were then placed in sterile tubes containing universal transport medium. The filled tubes were sealed and stored at a 4–6 °C cold chest before transport to the designated laboratory. Manufacturer-prescribed instructions were followed for sample preparation through the Nextractor NX-48 automated system and RT-qPCR steps to detect the same aforementioned gene targets (*RdRp*, *N*, *E* and human *RNase P*). The criteria for RT-qPCR run validity and NOS sample positivity were the same as those for saliva samples.

### Data analysis

The Buderer technique^10^ was used to compute minimum sample size. Presuming the prevalence of COVID-19 at 10% during the period of study planning, a target sensitivity and specificity of at least 90% for saliva-based RT-qPCR compared to NOS RT-qPCR, and the significance level (α) and maximum acceptable width of the 95% confidence interval (CI) being set at 0.05 and 10%, respectively, yielded a minimum sample size of 385.

Descriptive statistics were presented as proportions for categorical variables and median (interquartile range or IQR) for continuous variables. Diagnostic validity estimates (sensitivity, specificity, positive predictive value or PPV, negative predictive value or NPV, and accuracy) and their 95% CIs were calculated, using the MedCalc online calculator^11^, for three scenarios: (1) SalivaDirect RT-qPCR as index test and NOS RT-qPCR as reference test, (2) SE RT-qPCR as index test and NOS RT-qPCR as reference test, and (3) SalivaDirect RT-qPCR, SE RT-qPCR and NOS RT-qPCR as index tests separately benchmarked against a composite reference standard (CRS). In the CRS, a participant with at least either a SARS-CoV-2-detectable NOS or SalivaDirect or SE sample was considered positive and a volunteer with all samples without SARS-CoV-2 gene detection as negative for the virus^12^. Such a paradigm would generate perfect specificity and PPV for all index tests, since there would be no considered false positive results.

McNemar χ^2^ test was used to statistically compare the sensitivity, specificity and accuracy of SE RT-qPCR and SalivaDirect RT-qPCR against NOS RT-qPCR. To compare the PPVs and NPVs of the two saliva-based index tests, with the swab test as the reference standard, the weighted generalized score test statistic approach developed by Kosinski^13^ was utilized. Cohen κ coefficients for agreement between the designated index and reference tests were also estimated^14^. To assess statistical difference in sensitivity and accuracy, against the CRS, between NOS RT-qPCR, SE RT-qPCR and SalivaDirect RT-qPCR, Cochran Q omnibus test with McNemar χ^2^ test as pairwise comparison post hoc technique was implemented. In the case of the three-way comparison of resulting NPVs against the CRS, the aforementioned Kosinski approach was conducted pairwise with Bonferroni correction. An analysis comparing the C_q­_ values of SARS-CoV-2 genes and *RNase P* between valid NOS, SalivaDirect and SE samples among volunteers positive in at least one test was also conducted through the Skillings-Mack omnibus test^15^ with Wilcoxon signed-rank test as pairwise comparison post hoc technique. Statistical significance was set at p ≤ 0.0500 unless otherwise specified. The rest of statistical analyses were performed in Stata 14.2 (StataCorp LLC, College Station, Texas, United States of America).

## Results

**Figure 1:**
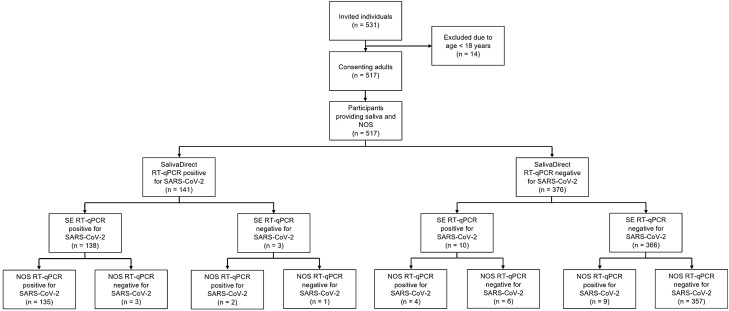
Flow diagram of participants in the study. *NOS* naso-oropharyngeal swab, *RT-qPCR* reverse transcription quantitative/real-time polymerase chain reaction, *SARS-CoV-2* severe acute respiratory syndrome coronavirus 2, *SE* saliva with RNA extraction.

A total of 517 adults provided paired NOS and saliva samples for SARS-CoV-2 testing (Fig. 1). The median (IQR) age of the entire sample was 31 (26–43) years and 224 (43.33%) were female. Majority of the participants were outpatients (n = 474, 91.68%) and asymptomatic (n = 387, 74.85%). Among participants who were symptomatic (n = 130, 25.15%), the median (IQR) time from reported symptom onset to sample collection was 5 (2–8) days. A total of 150 (29.01%) NOS and 151 (29.21%) saliva samples were positive for SARS-CoV-2, while 160 (30.95%) participants had at least one positive sample. Among those with a positive saliva sample, SARS-CoV-2 was detected in 148 (28.63%), 141 (27.27%) and 138 (26.69%) participants whose specimens were processed under SE, SalivaDirect and both approaches, respectively. There were nine (1.74%) participants with NOS positive and saliva negative for SARS-CoV-2, and another ten (1.93%) whose saliva samples were positive for the virus while their NOSs were negative. In the latter subset of volunteers, three gave samples that were positive in both SE RT-qPCR and SalivaDirect RT-qPCR, six were positive only in SE RT-qPCR and one was positive only in SalivaDirect RT-qPCR.

The resulting contingency table comparing SE RT-qPCR with conventional NOS RT-qPCR as reference (Table 1) revealed sensitivity of 92.67% (95% CI 87.26%, 96.28%), specificity of 97.55% (95% CI 95.40%, 98.87%), PPV of 93.92% (95% CI 89.00%, 96.72%), NPV of 97.02% (95% CI 94.85%, 98.29%) and accuracy of 96.13% (95% CI 94.09%, 97.62%). On the other hand, comparing SalivaDirect RT-qPCR to the swab-based test yielded sensitivity of 91.33% (95% CI 85.64%, 95.30%), specificity of 98.91% (95% CI 97.23%, 99.70%), PPV of 97.16% (95% CI 92.81%, 98.91%), NPV of 96.54% (95% CI 94.32%, 97.91%) and accuracy of 96.71% (95% CI 94.79%, 98.07%). There appeared no difference between the two sensitivity (McNemar χ^2^ [degrees of freedom or df = 1] = 0.6670, p = 0.4142), specificity (McNemar χ^2^ [df = 1] = 3.5714 p = 0.0588), accuracy (McNemar χ^2^ [df = 1] = 0.6923, p = 0.4054), PPV (Standard χ^2^ [df = 1] = 0.8173, p = 0.3660) and NPV (Standard χ^2^ [df = 1] < 0 0.0001, p > 0.9920) estimates. The Cohen κ coefficient estimate for agreement between the SE and swab tests is 0.82 (95% CI 0.75, 0.90) while that between the SalivaDirect and swab tests is 0.85 (95% CI 0.78, 0.92), both suggesting substantial agreement.

**Table 1:** Diagnostic agreement contingency table and estimates comparing SE and SalivaDirect RT-qPCR, as separate index tests, to NOS RT-qPCR for SARS-CoV-2 detection.

NOS RT-qPCR	SE RT-qPCR				TOTAL
	Positive		Negative		
	SalivaDirect RT-qPCR		SalivaDirect RT-qPCR		
	Positive	Negative	Positive	Negative	
Positive	135	4	2	9	150
Negative	3	6	1	357	367
Total	138	10	3	366	517

*NOS* naso-oropharyngeal swab, *RT-qPCR* reverse transcription quantitative/real-time polymerase chain reaction, *SE* saliva with RNA extraction.

When NOS RT-qPCR, SE RT-qPCR and SalivaDirect RT-qPCR were individually compared to the CRS (Table 2), the sensitivity estimates were 93.75% (95% CI 88.81%, 96.96%), 92.50% (95% CI 87.27%, 96.06%), and 88.13% (95% CI 82.08%, 92.70%), respectively. No statistically significant difference was found between these values (Cochran Q [df = 2] = 5.3600, p = 0.0686). Accuracy was also similar across the tests in this paradigm and was estimated to be 98.07% (95% CI 96.47%, 99.07%), 97.68% (95% CI 95.98%, 98.80%) and 96.32% (95% CI 94.32%, 97.77%) for NOS RT-qPCR, SE RT-qPCR and SalivaDirect RT-qPCR, respectively. The three NPVs from these tabulations were also not different from each other (for all pairwise comparison findings: standard χ^2^ [df = 1] < 0.0001, p > 0.9920).

**Table 2:** Diagnostic validity contingency table and parameter estimates comparing NOS RT-qPCR, SE RT-qPCR and SalivaDirect RT-qPCR as separate index tests to the CRS for SARS-CoV-2 detection.

CRS	NOS RT-qPCR								Total
	Positive				Negative				
	SE RT-qPCR				SE RT-qPCR				
	Positive		Negative		Positive		Negative		
	SalivaDirect RT-qPCR		SalivaDirect RT-qPCR		SalivaDirect RT-qPCR		SalivaDirect RT-qPCR		
	Positive	Negative	Positive	Negative	Positive	Negative	Positive	Negative	
Positive	135	4	2	9	3	6	1	0	160
Negative	0	0	0	0	0	0	0	357	357
Total	135	4	2	9	3	6	1	357	517

*CRS* composite reference standard, *NOS* naso-oropharyngeal swab, *RT-qPCR* reverse transcription quantitative/real-time polymerase chain reaction, *SE* saliva with RNA extraction.

**Figure 2:**
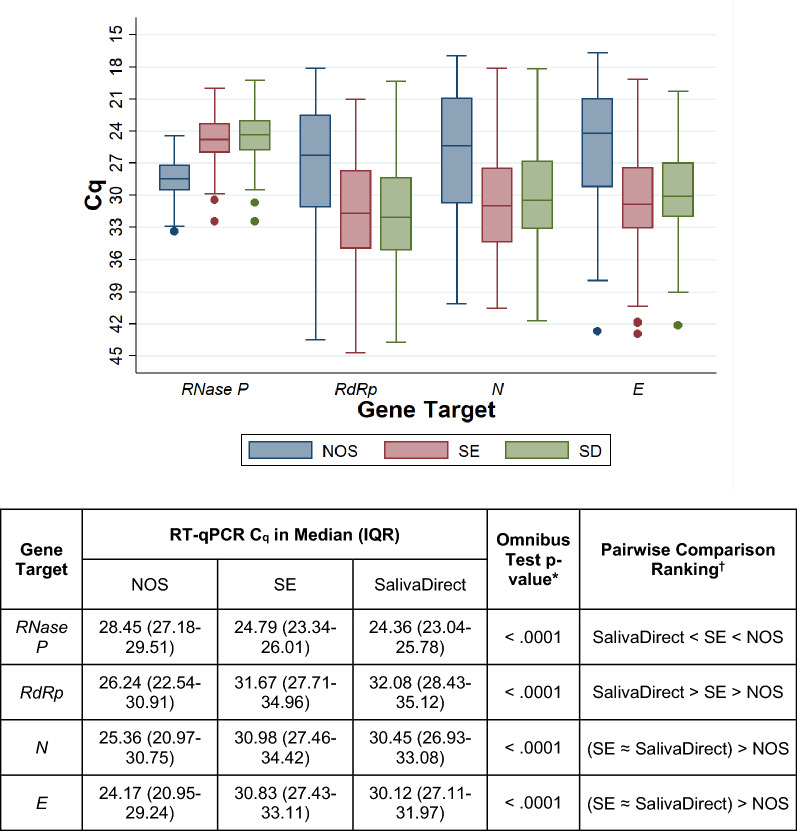
RT-qPCR C_q_ values from NOS, SE and SalivaDirect sample trios among study volunteers with positive reading on at least one test (n = 160). *C*_*q*_ quantification cycle, *E* envelope, *IQR* interquartile range, *N* nucleocapsid, *NOS* naso-oropharyngeal swab, *RdRp* RNA-dependent RNA polymerase, RT-qPCR reverse transcription quantitative/real-time polymerase chain reaction, *SE* saliva with RNA extraction. *Through Skillings-Mack test. ^**†**^Through Wilcoxon signed-rank test with Bonferroni correction (adjusted threshold for statistical significance: p_corrected_ ≤ .0167).

In the participant subset with at least one valid sample positive for SARS-CoV-2 (n = 160, 30.95%), the C_q_ readings for each SARS-CoV-2 (*RdRp*, *N* and *E*) and internal control (*RNase P*) gene targets were also compared across the three testing modalities (Fig. 2). Skillings-Mack omnibus tests yielded statistically significant global differences in C_q_ readings between samples subjected to NOS RT-qPCR, SE RT-qPCR and SalivaDirect RT-qPCR in all genes of interest (for all target genes: p < 0.0001). Greater SARS-CoV-2 target gene copies (lower C_q_ readings) were generally found in NOS samples compared to SE and SalivaDirect samples, while copies of human *RNase P* appeared to be more abundant in saliva, regardless of processing, than in NOS.

## Discussion

Substantial evidence already exists on the non-inferiority of saliva relative to NOS as a specimen for conventional RNA extraction-dependent RT-qPCR detection of SARS-CoV-2^7,8^. Compared to NOS as a testing sample, the use of saliva has several advantages^16^. In this alternative setup, compared to the number of staff that must be trained for adequate-quality NOS collection one patient at a time, fewer personnel needing less technical training are needed to facilitate simultaneous self-collection of saliva by multiple patients. This potentially translates to a less aggregate risk of SARS-CoV-2 transmission between patients and personnel (due to avoidance of close-contact interactions secondary to swabbing, and fewer personnel contact with materials that could not be sanitized beforehand such as patient samples) and reduced demand for personal protective equipment (PPE). In terms of logistics, storage of samples before transport to the laboratory is less complicated (with the use of plain sterile vials rather than a combination of tubes, ice-cooled chest boxes, and proprietary swabs and transport reagents). Patients are also expected to be more amenable to such sample collection for SARS-CoV-2 testing than invasive and uncomfortable swabbing. It is also important to note that variation of several patient and personnel factors related to naso-oropharyngeal swabbing introduces iatrogenic preanalytical variability that may adversely affect testing results^17,18^. Furthermore, these findings may be useful in light of recent reports suggesting changing patterns of viral shedding, tissue tropism, and transmission mechanics^19–21^, in favor of oral cavity and saliva, depending on the emerging SARS-CoV-2 variant such as Omicron^22^.

Nevertheless, there remains fewer endeavors on attempting to streamline these laboratory processes without significant decline in analytical and/or diagnostic performance^9,23^. This is particularly important in the geographic and sociopolitical contexts of developing countries. Such settings are associated with challenges in addressing demands for COVID-19 pandemic response with meager resources, such as sustaining testing coverage that is accurate, adequate and timely^24^. In the Philippines, a previous undertaking^25^ adapted the RNA extraction-free technique, known as covidSHIELD, of the University of Illinois at Urbana-Champaign^23^ for saliva RT-qPCR detection of SARS-CoV-2. This has led to the first regulatory approval of the technology in the country through Sansure Biotech-based molecular biology laboratory facilities of the Philippine Red Cross^26^. The present study, on the other hand, sought to evaluate the SalivaDirect procedure^9^ through the harmonized laboratory system of a private hospital network, which utilizes GeneFinder reagents and the Bio-Rad RT-qPCR platform. While it may appear that covidSHIELD involves theoretically cheaper reagents for saliva processing (Tween 20 and Tris–Borate-Ethylenediaminetetraacetic acid [TBE] buffer) compared to SalivaDirect’s Proteinase K, an advantage of the latter is that its heat-inactivation step is only 5 min or one-sixth of the time required by the former. On top of the aforementioned benefits of the testing regime shift, from swab-based to saliva-based, streamlined pre-RT-qPCR saliva processing through heat inactivation and Proteinase K addition further reduces (1) the resource cost to release SARS-CoV-2 RNA copies from capsids compared to the use of proprietary reagents, and (2) the risk of laboratory personnel exposure to viable SARS-CoV-2 units in patient samples (as the virus is known to be denatured at a fraction of time and thermal energy applied during the prescribed inactivation step^27^).

The results of the present study add to the growing body of evidence that indicate favorable comparability of saliva and NOS as specimens for SARS-CoV-2 nucleic acid-based detection. The head-on comparisons between SE RT-qPCR and NOS RT-qPCR and between SalivaDirect RT-qPCR and NOS RT-qPCR reveal statistically similar diagnostic accuracy parameter estimates. Furthermore, our analyses showed that among volunteers positive in at least one of the three tests, the aggregate C_q_ values for two SARS-CoV-2 gene targets (*N* and *E*) were not significantly different between paired SE and SalivaDirect specimens while the average *RdRp* C_q_ in SE specimens was statistically higher, albeit minimally so, than in those processed through SalivaDirect. As the C_q_ for a genomic target is inversely proportional to the initial amount of target gene copies in the post-processed mixture to be subjected to RT-qPCR, these findings imply that the presumed increase in heterogeneity of heat-inactivated and Proteinase K-treated saliva, compared to saliva that was processed conventionally, did not substantially affect the analytical, diagnostic and quantitative performance of the RT-qPCR procedure common and downstream to both tests. However, compared to counterpart values for NOS, the SARS-CoV-2 viral load and the human cell content appear to be lower and higher, respectively, in both saliva specimen types. Higher *RdRp*, *N* and *E* C_q_ readings for NOS than both SE and SalivaDirect suggest that either (1) the viral load in the nasopharyngeal area is higher than in the oral cavity or (2) the liquid volume of saliva led to a “dilution” of the corresponding SARS-CoV-2 content. The significant discrepancy in human *RNase P* C_q_ values may be due to the higher human cell content in saliva than in the nasopharyngeal swab scrapings.

Because volunteers having a positive saliva test result and a negative swab test result will be considered false positives in head-on comparison (with NOS RT-qPCR as the reference standard), the estimated specificity and PPV would have appeared to be lower than what could be expected in an RT-qPCR platform. Assuming that optimal quality control procedures were observed from test sample collection to RT-qPCR, specificity and PPV are theoretically 100% (making it impossible to assign a sample as a false positive) in RT-qPCR because the primers designed to detect the genetic material of a pathogen is highly specific, to molecular regions in its genomic sequence^2^. It is thus more likely that saliva-positive but swab-negative volunteers represent truly-infected cases missed by swab testing, rather than false positives, and vice versa. Arguably, a false sense of security may befall such patients if they were tested only by NOS RT-qPCR. We hypothesize that this subset of the population may be a greater clinical or public health risk than their swab-positive but saliva-negative counterparts, considering that SARS-CoV-2, in terms of anatomy and biomechanics, is more likely to be released from the host to the environment (through aerosolization and droplet formation) in saliva than in the nasopharynx^16,28^. With this consideration of perfect specificity and PPV in mind, we then analytically constructed a CRS wherein the criterion for a positive case is at least either a SARS-CoV-2-positive NOS, SE or SalivaDirect sample (virtually ensuring specificity and PPV of 100% since no volunteer can be considered false positive), and that for a negative case is SARS-CoV-2-negative NOS, SE and SalivaDirect samples. With this benchmark, the diagnostic performance of NOS RT-qPCR, SE RT-qPCR and SalivaDirect RT-qPCR was separately assessed. All tests performed statistically similar to one another in terms of sensitivity, NPV and accuracy in this scenario, again suggesting that both saliva as a sample and the streamlined technique to handle this specimen are non-inferior, at the very least, to the conventional swab test in detecting SARS-CoV-2. Another remarkable finding that is apparent in this construction is that all tests, even NOS RT-qPCR, which is the considered reference standard in practice, was not able to detect SARS-CoV-2 in some volunteers whose saliva samples turned positive (n = 10). While this can be due to iatrogenic factors (e.g., patient-specific and personnel-specific aspects concerning naso-oropharyngeal swabbing quality), potential pathophysiological and fluid-tissue localization mechanisms that are yet to be elucidated in SARS-CoV-2 infections may also be at play. As saliva testing also missed a small subset of individuals with positive swab results (n = 9), there is perhaps some epidemiological value in utilizing more than one sample type to detect SARS-CoV-2 infection. The study was conducted during a period (May to December 2021) before community transmission of the Omicron variant which has demonstrated greater salivary tropism than earlier-circulating variants of concern (such as Alpha, Beta and Delta), was confirmed locally^29^. Thus, the possibility of saliva-based testing performing better than the NOS-based counterpart during surges of Omicron and other or future Omicron-like variants (that replicate better in anatomic areas that produce or are supplied with saliva) cannot be disregarded.

We recognize several limitations of this study. As a cross-sectional design was implemented, follow-up through clinical assessment and laboratory testing for at least one time point after initial study contact was not performed. Such a procedure could demonstrate viral kinetics in the specimens through the preclinical, clinical, and convalescent or mortality phases of the infection. This study was also insufficiently powered to determine the impact of sample collection timing (from the history of COVID-19 exposure and/or onset of relevant symptoms) on test results. RT-qPCR of NOS samples that underwent RNA extraction-free processing was also not performed; this would have provided direct information on the difference in categorical and quantitative (C_q_) results due to the change in pre-RT-qPCR processing technique for swab specimens. It will also be interesting to comprehensively assess contextual differences between individuals with swab-positive/saliva-negative and swab-negative/saliva-positive results. We also suggest that head-on comparison of covidSHIELD and SalivaDirect be conducted to generate primary evidence on the relative performance and utility of these two major streamlining approaches for saliva sample pre-RT-qPCR processing. In this regard, a recent study that further streamlined SalivaDirect, by removing the proteinase K addition step and adding heat pretreatment, showed preservation of viral detection and assay sensitivity performance^30^. As these SalivaDirect modifications are highly reminiscent of covidSHIELD, this is a possible indication that the two technologies have similar, if not equivalent, diagnostic accuracy.

## Data Availability

The corresponding author can provide the data upon request. It was submitted as part of the terminal report to the Medical Center Manila Ethics Review Board.
